# Systems leadership in practice: thematic insights from three public health case studies

**DOI:** 10.1186/s12889-020-09641-1

**Published:** 2020-11-17

**Authors:** Charlotte Bigland, David Evans, Richard Bolden, Maggie Rae

**Affiliations:** 1 Public Health England South West, 3rd Floor, 2 Rivergate, Bristol, BS1 6EH UK; 2 Centre for Public Health and Wellbeing, University of the West of England, Bristol, BS16 1QY UK; 3 Bristol Leadership and Change Centre, Bristol Business School, University of the West of England, Bristol, BS16 1QY UK; 4 Visiting Professor Public Health Centre for Public Health and Wellbeing, University of the West of England, Bristol, BS16 1QY UK

**Keywords:** Public health, Systems leadership, Health protection, Healthcare public health, Health improvement, Leadership

## Abstract

**Background:**

‘Systems leadership’ has emerged as a key concept in global public health alongside such related concepts as ‘systems thinking’ and ‘whole systems approaches.’ It is an approach that is well suited to issues that require collective action, where no single organisation can control the outcomes. While there is a growing literature on the theory of systems leadership in a number of fields, there remains a lack of published empirical studies of public health systems leadership for professionals to learn from. The aim of the current project was to conduct cases studies in UK public health to provide empirical evidence on the nature of effective systems leadership practice.

**Methods:**

Three system leadership case studies were identified in the key domains of public health: health protection, healthcare public health and health improvement. A total of 27 semi-structured interviews were conducted. Data were thematically analysed to identify the components of effective systems leadership in each case and its impact.

**Results:**

The thematic analysis identified themes around ‘getting started,’ ‘maintaining momentum’ and ‘indicators of success’ in systems leadership. In terms of getting started, the analysis showed that both a compelling ‘call to action’ and assembling an effective ‘coalition of the willing’ are important. To maintain momentum, the analysis identified themes relating to system structure, culture and the people involved. Regarding culture, the main themes that emerged were the importance of nurturing strong relationships, curiosity and a desire to understand the system, and promoting resilience. The analysis identified three components that could be used as indicators of success; these were a sense of enjoyment from the work, resource gains to the system and shifts in data indicators at the population level.

**Conclusions:**

This study has provided insight into the nature of systems leadership in public health settings in the UK. It has identified factors that contribute to effective public health systems leadership and offers a thematic model in terms of establishing a systems leadership approach, maintaining momentum and identifying key success indicators.

## Background

‘Systems leadership’ has emerged as a key concept in global public health alongside such related concepts as ‘systems thinking’, where emphasis is place on the relationships between the parts that form a physical system in addition to understanding the individual parts and their environment separately [1], and ‘whole systems approaches’ [1]. Systems leadership is an approach that is well suited to issues that require collective action and has been applied to a number of public health issues including obesity [2, 3] and sustainable development [4]. In the UK, Public Health England (PHE) recently commissioned a scoping report on how to develop public health systems leaders [5] as there is a concern that current training might not fully prepare public health professionals for these roles. This report identified that while there is a growing body of literature on the theory of systems leadership in a number of fields, there is a lack of published empirical studies of public health systems leadership for professionals to learn from. The aim of the current project was to conduct a number of case studies in UK public health and thematically analyse the findings to produce empirical evidence on the nature of effective systems leadership in practice.

### What is systems leadership?

Where systems leadership differs most significantly from older leadership concepts is through the focus on leading beyond organisational and professional boundaries in order to address cross-cutting ‘wicked’ problems [6, 7]. However, despite increased recognition, there is no commonly agreed definition of systems leadership. In a health and wellbeing context, the UK NHS Confederation has described it as follows:

“System leadership is about local leaders from across the health and care system sharing a cohesive approach to working together to improve the local health and care system … System leaders have clear, shared priorities that are grounded in the needs of their communities and not in the interests of individuals or their organisation.” [8]

A more general definition was offered by Ghate et al. [9] in a study on systems leadership in public services although this is compatible with systems leadership in any setting where professionals are grappling with volatile, uncertain, complex and ambiguous (VUCA) contexts [10]. Ghate et al. [9] describe systems leadership as having two key characteristics:

(a) “it is a collective form of leadership” concerned with “the concerted effort of many people working together at different places in the system and at different levels,” and

(b) it “crosses boundaries, both physical and virtual, existing simultaneously in multiple dimensions.”

Both definitions stress that the leadership required transcends a single organisation and is a collective effort. The framework arising from this work demonstrates how systems leadership requires a focus not just on the characteristics of individual systems leaders, but also the organisational contexts in which they operate, the wider public service context and the needs and outcomes of service users [9].

### Current evidence around systems leadership in UK public health practice

Since the 2013 re-organisation of public health in England there have been five major studies of aspects of the UK public health system. One study was a comparison of public health in the four UK nations [11] and four focused on aspects of the public health system in England [12–15]. None of these focused exclusively on systems leadership in public health, but all referred to it either explicitly, or in some cases implicitly when discussing ‘influencing’.

A literature search did not identify any detailed empirical studies of effective systems leadership in UK public health. Instead, its absence was noted with one report, concluding that there was “little evidence of HWBs [Health and Wellbeing Boards] providing genuine systems leadership across the piece” [13].

Whilst little relevant empirical research on systems *leadership* in public health was identified, there have been several recent review papers of the related concept of systems *thinking* in public health. Carey et al. [16] published a systematic review on systems science and systems thinking for public health, identifying 117 articles which they grouped into four categories. The majority of the sources were editorials, commentaries or reviews, or research papers that used systems as a broad analytical lens. While some mentioned systems leadership, none provided detailed evidence on what it involved in practice.

Overall, the editorial, commentary and theoretical literature on systems leadership in public health far outweighs the empirical research literature. In addition, this literature focuses more on systems thinking and approaches, rather than systems leadership per se. The limited systems leadership literature identified focuses on *what* systems leadership is, or *why* it is important rather than *how* it is accomplished in practice. This study seeks to address this identified gap in the literature by reporting on three empirical case studies of systems leadership in UK public health practice.

## Methods

### Design and approach

This study used qualitative case studies [17] to explore the phenomenon of systems leadership in UK public health settings. Thematic content analysis was applied to identify common themes in data from semi-structured interviews [18]. An interdisciplinary approach was taken with two of the researchers being experienced public health professionals (CB and MR), one a health services researcher (DE) and one an expert on systems leadership (RB).

### Case Study Selection and Recruitment of Interviewees.

A call for case study abstracts was circulated between August and September 2019 to members of 11 pan-UK public health organisations and fora. Members were also asked to cascade the request to their professional networks.

Twenty-two descriptive case study submissions that outlined the projects were received of which 18 contained enough detail to be taken through a formal screening process and scored using criteria developed from Ghate et al. [9]. Using the Ghate definition described in the introduction and adding the element of courage/innovation described by others in the field [8], submissions were scored against evidence of 1) **“**VUCA” environment, 2) having outcomes for services users at its heart, 3) cross boundary working with multiple organisations, cultures or geographies 4) no one organisation having exclusive command and control power over the system 5) a sense of collective endeavour and 6) innovation/paradigm shift/risk taking. Only those that demonstrated evidence of all six components were taken forward. Six cases studies met all the above criteria. From these six, three case studies were selected to represent the breadth of public health practice. One case study from each of the three pillars of public health (health protection, healthcare public health and health improvement) was selected and the cases covered a range of geographies and population demographic in England.

Following selection, the primary contact for each case study was asked to suggest key interviewees. To ensure a diverse range of perspectives, both public health and non-public health professionals were selected. In addition to the case study interviews, a senior national or regional public health leader from each domain of public health was also interviewed. These interviews were used to provide a broader background perspective on systems leadership in public health to complement the cases study interviews.

### Data collection

Potential participants were contacted by email and invited to take part in an approximately one-hour interview. 30 individuals were approached of which 27 agreed to be interviewed (the other three, one from each case study, were unavailable within the timescales). Interviews were semi-structured in format and used a standard interview topic guide designed for this study. The guide is available as supplementary information as Additional File 1. The guide was piloted within the project team and with a participant from the Action on ACEs (adverse childhood experiences) case study. The interview topic guide provided prompts to cover background information on the interviewee and the project, the interviewee’s personal attributes and leadership style, information on relationships, power, risks/innovations and whether conflict was experienced. The interviewee’s views on the key barriers and enablers experienced and whether aspects discussed changed with time were also explored.

Fourteen of the interviews were face-to-face and thirteen were by telephone. All interviews were audio-recorded with participants providing oral consent before starting. Audio recordings were transcribed by a professional transcription service. Transcripts were checked against recordings for accuracy and corrected as needed. The majority of interviews lasted approximately 50 min with the longest being 1 h 5 min and the shortest 28 min. An overview of each case study and its interviewees is given in Table 1.

**Table 1:** Descriptive overview of the case studies and their interviewees

Case Study				Background Expert Interviewees
	Action on ACEs Gloucestershire	Cheshire and Merseyside Blood Pressure Partnership Board	Wiltshire Novichok poisoning response and recovery	
**Brief Description**	ACEs are adverse childhood experiences or traumatic events that happen in childhood and can go on to impact later life. Action on ACEs Gloucestershire’s mission is to build communities and organisations that are aware of ACEs, talk about ACEs and take action on ACES. It involves multiple partners from across the public and third sector. Commenced 2017.	Prolonged high blood pressure (BP) can have a number of adverse health outcomes. This work aims to reduce the impact of high BP across the sub region of Cheshire and Merseyside. Remit includes prevention, detection and management of high blood pressure. Multiple partners from public, private and third sector. Commenced 2013.	Novichok is a harmful nerve agent that was found at sites in Wiltshire in 2019. Five people were contaminated at a toxic level during two distinct but related incidents. Many more people across Wiltshire had their lives severely impacted. The multi-agency tactical and strategic control groups were in operation between spring and summer 2019.	Experienced public health consultants with a national and/or regional profile in their area of public health activity
**Area of focus**	Health improvement	Healthcare public health	Health protection	All
**Interviewees**	A1-A7	B1-B11	N1-N7	E1-E3
**Number interviewed from each profession**	Local Authority Public Health: 3 Police: 2 Housing Association: 1 Education: 1	Fire and Rescue: 1 Public Health: 5 Voluntary and Community Sector: 1 Pharmacy: 1 Lay member: 1 National Health Service (NHS): 1 Local Councillor: 1	Public Health England: 2 Local Authority Public Health: 3 Police: 1 Local Authority: 1	Health protection: 1 Healthcare public health/population health: 1 Health improvement: 1

Transcripts were returned to interviewees to check for accuracy. Three interviewees made minor edits to clarify meaning, and two asked to remove small sections that contained sensitive material.

### Data analysis

NVivo 12 software was used to facilitate analysis. All transcripts were initially coded by CB with DE independently coding a subset. In line with best practice outlined in Braun and Clarke [18] multiple initial data codes were generated and then the data codes were sorted and analysed to identify sub and main themes. The initial data codes were a combination of theory-driven deductive codes derived from existing literature, and data-driven inductive codes. The initial deductive codes drew on work from the Kings Fund [19–21], a well-respected UK think tank focusing on public health and leadership issues, as well as other studies of systems leadership in the public sector more broadly [5, 9]. The themes identified were reviewed, and further defined and refined by CB and DE initially, and then by the full project team until a final consensus was reached. When analysing the data, areas of similarity as well as areas of difference were explored.

## Results

Twenty seven individuals were interviewed to gain background information and data for the three case studies. Table one summarises the nature of each case study and the range of interviewees.

The thematic analysis resulted in characterising themes around ‘getting started,’ ‘maintaining momentum’ and ‘indicators of success’ in systems leadership. The major themes, along with their primary and secondary sub themes are summarised in Table 2.

**Table 2:** Themes and sub-themes identified

Theme	Primary Sub Theme	Secondary Sub Themes		
1. Getting started	a. Call to action			
	b. Assembling the coalition of the willing			
2. Maintaining momentum	a. Structure	Governance	Resources	
	b. Culture	Relationships	Curiosity	Resilience
	c. People	Personal characteristics	Values	
	d. Paradoxes	Power	Conflict	Uncertainty
3. Indicators of success	a. Sense of enjoyment and shared endeavour			
	b. Increased momentum with gains to system			
	c. Shifts in evaluation metrics			

**Fig. 1:**
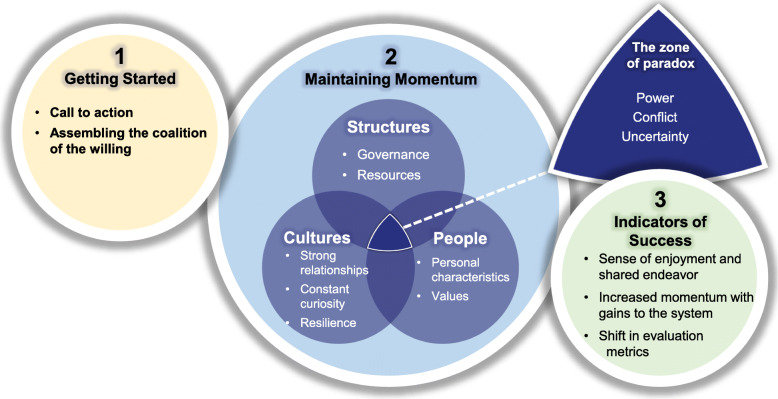
The interplay of the systems leadership themes identified from the three cases studies. Image created for this study by the authors

Many of these themes and subthemes were found to be interconnected and a simplified overview of this is given in Fig. 1.

### Getting started

Issues that require a systems approach are complex without a clear solution, thus often:

“The hard bit is getting it started.” (B10).

#### Call to action

Getting started in the three systems focussed around “going with the energy” (B10). In each case there was a call to action message and the assembly of an effective coalition of the willing to drive system change. The call to action was most compelling in the Novichok situation where the system was brought together by a legally mandated process following declaration of a health protection incident. In all cases the effective call to action message resonated with a wide cross section of its target audience, not just the public health community.

“How can you argue against the values of it, how can you, how can anybody with any credibility say this is a bad idea?” (A7)

The importance of a *credible* message as a call to action was emphasised by one senior interviewee who stated:

“There’s an element of people will focus on something and will put an effort in when … they actually believe the message is true.” (B10)

The interviews illustrated that there was not always a single, narrow, interpretation of the call to action message.

“We have a constant overarching aim, but everyone’s rationale for being involved varies, and we just have to run with that.” (B3)

#### Assembling the ‘coalition of the willing’

The second theme identified in getting started was assembling the ‘coalition of the willing.’ In all three case studies this was done using set piece events where the call to action was shared. For the Action on ACEs and Cheshire and Merseyside blood pressure (BP) work this involved workshops and conference style events, for Novichok it involved the assembly of the incident management team.

“We started off with a very normal approach of a workshop, key speakers, present an issue, hold the mirror up to the system… and it had the best effect I’ve ever seen. I’ve never seen a reaction like it.” (A3)

Gathering appropriate and receptive stakeholders was identified as important in all the case studies.

“There were colleagues from all sort of different partners who hadn’t been involved in blood pressure. For them it was the first time [they heard the issues] and they … sat at our table and said, ‘this means something to us’.” (B5)

Both the Novichok and Cheshire and Merseyside BP case studies identified that preparing system leaders to work together ahead of project launch was important.

“I cannot overemphasise how important [incident response] exercising is. …if you work with people and practise then exercise with them… it does become that sort of psychological known situation” (E3)

A number of interviewees acknowledged that timing and luck played a part getting started, with one interviewee describing the need to “create the conditions for serendipity” (E1).

### Maintaining momentum

This section explores a number of facets of systems leadership involved in sustaining change. While they are described sequentially, in reality the structure, culture and people components interact with each other concurrently, and all have a part in shaping the systems leadership process and outcomes.

#### Structure

This section summarises the themes around governance and authorising structures, and the resources or operating capacity available to the system.

##### Governance

An expert interviewee postulated that effective governance in a system is dynamic and requires calibration to that system’s needs at a set point in time.

“There are only guide rails because there are no cookbooks for it…holding that [effective governance] position is a cycle.” (E2)

In all three case studies interviewees recognised the need for calibration of the governance structures and cited examples of where change and adaptation had occurred.

“There is a need to strike a balance for your system, to understand when some governance is needed and when you need to let the chaos run free.” (A3)

“They created what became known as the … health emergency response cell (HERC). [It] didn’t create the right links at a local level initially… we just couldn’t have two parallel processes, so we changed it.” (N4)

In the ACEs and BP case studies, the governance structures started out loose but with time and changing circumstance became tighter. In Novichok the initial governance started out as quite prescriptive but adjusted to suit the specific situation. It was acknowledged that calibrating governance appropriately is complex and iterative.

##### Resources

In all three cases studies the key resources were people and their invested time, along with cash funding. Having a dedicated coordinator was seen as crucial.

In the early phases, interviewees referred to the lack of money as a positive as it made people concentrate on what else they had. The most commonly identified compensatory resource was people and their time.

“That [funding request] was unsuccessful, which was probably the best thing that happened because it made us… realise that we had to do something without money.” (A4)

It was clear from the interviews that limited funds were only facilitatory up to a certain point.

“We had some difficult discussions about how you chunk up how much everybody should pay. It felt quite fragile at times, that it could … potentially pull us apart, but we found a way.” (B2)

In the longer term, securing funding was seen as important, both directly as a way to finance work, but also as a signal of confidence in the process.

“Well, a little bit of money can make a real difference to be honest with you, and I think that what [name of organisation] did in terms of securing the [£100K] funding was absolutely key to the success of the work that we’re talking about today.” (B1)

Having a dedicated, central co-ordinator was identified as a key resource to maintain momentum. Across the case studies the seniority and precise activities of the coordinator varied, but the importance of the coordinator was stressed explicitly in all three case studies.

“You can’t have a system without a coordinator…. I think for every system there needs to be a coordinator, or a coordinating team.” (B5)

#### Culture

This section considers system culture and the behaviours that underpin it. Three key areas were identified from the interviews: relationships, curiosity and resilience.

##### Relationships

The central importance of relationships was recognised in all case studies, with interviewees emphasising that good relationships are essential to effective systems leadership.

“It’s *all* about relationships, … and the more complicated the system is, the more important it is that people have relationships” (B10).

***Forming relationships***

How relationships were formed varied and was dependent on the personalities involved. Some interviewees felt forming relationships came naturally while others described having to consciously work on nurturing relationships.

“I'm an extreme introvert… I don’t naturally do networking, I hate networking, but I do know that I need to have really good relationships.” (N7)

Whether relationships had been formed pre or post working on the specific initiative varied with both being described as useful. A further dichotomy was whether people chose to develop relationships at a professional or at a more personal level. In Novichok it was acknowledged that there was little time to dedicate specifically to relationship building although the intensity of the situation did produce strong relationships quite rapidly. In contrast, in ACEs doing this was built into the behaviours adopted by individuals, and in the Cheshire and Merseyside BP work this was built structurally into practice.

“We work on it [building personal relationships] and so, for example, we start every meeting with good news and gratitude ... And there’s a relationship between the directors - they know about each other and their families. And there are the development days we have done to really build that bond and those relationships, and to help them function.” (B2)

***Trust between system leaders***

Reciprocal trust was identified as important in nurturing robust relationships able to overcome the expected frustrations that come with systems working.

“If you know the human it allows you to build trust, and with trust comes tolerance, and humans are annoying, and humans niggle a bit, but if you built up that level of trust you have tolerance ….and you get a positive vibe.” (A5).

Demonstrating that system partners were both valued and respected contributed to building trusted relationships. Interviewees described both the behaviours they used to demonstrate they valued colleagues and what made them feel valued. There was a good correlation between the two.

“We don’t learn from just being constantly criticised, we need to think praise as well. If someone’s done something great I’ll send them a note and say thank you, buy them flowers, buy them chocolates. Whatever is their thing.” (E3)

There was also a correlation between what people did to show respect and what made people feel respected.

“I think that professional respect shown by our strategic coordinating group leader for what we were bringing to the table just helped ensure that rank didn’t really interfere with what it was we were trying to do.” (N4)

While not an absolute divide, behaviours that were associated with establishing a sense of feeling valued often focussed on a colleague and identifying ‘their thing’ at the personal level, while those associated with respect tended to focus on the professional and their expertise.

***Inclusive, safe environments***

Interviewees discussed the importance of creating a system culture of trust and safety. Behaviours cited as contributing to this included demonstrating personal vulnerability was acceptable, ensuring language was inclusive, and creating a culture where people felt safe to express contrary opinions.

“There was the type of systems leadership where leaders showed their belly and showed their vulnerability … but that they would do absolutely everything within the remit of their role to work with everybody else to get a good result….It completely empowers the whole room to feel that they can speak up and feel they can be a part of something … I think the sessions where we had individuals that didn't show vulnerability … well you didn't get as much out of it because people automatically shut down.” (N3)

The cases studies described positively the high levels of trust which were established within their systems. However, a potential downside of this was identified in terms of the balance that needed to be struck between serving “club and country” (E1).

“The individual agencies can say ‘oh, you have gone native’. You have gone native because the levels of trust can build up in the system at a higher level than they exist in your own organisation and that’s actually a threat.” (A5)

##### Curiosity

A focus on curiosity and seeking to understand the system was identified as a behaviour that the interviewees prioritised.

***Open mind-set***

Interviewees described that being open-minded was essential to successful systems leadership. This open mind-set was described in several different ways.

“So, for me it's something about recognising there's more than one way to do this, being open to some challenge from somebody else who might have a different view about it, or a different way through something.” (N1)

***The art of listening***

As well as being open minded to different perspectives, interviewees also described actively seeking information. The most frequently cited strategy was asking questions and then really listening to the answers.

“We keep on asking constantly, and it slows the process down, it massively slows the process down … I think I listen more than talk” (B3)

“Really good active listening is key.” (N3)

***Seeking understanding***

Interviewees took a structured approach to ensuring they explored information from multiple angles. No one said this was something that came intuitively in the way many interviewees did around themes like forming relationships. Instead interviewees talked in terms of having specific frameworks, suggesting it was a conscious process. Several different processing frameworks were described. A common framework was moving between strategic, tactical and operational perspectives.

“So, I've got a model in my head as I sit here. I think in four tiers or stratifications: vision, strategy, operations, tactics …You’ve got to be intellectually capable of just migrating up and down that within a minute, all through the day and night.” (N7)

It was acknowledged that “it’s quite difficult to see the experiences of others” (E3). Some interviewees suggested that this ability to move between perspectives might be more challenging in situations when professionals feel they are operating in their area of expertise.

“I think sometimes what I observe in people who are subject matter experts is that they get crosser and crosser that the rest of us can't see what they can see, but obviously we don’t have the same access to knowledge or experience that they’ve got in a particular subject.” (N1)

##### Resilience

The importance of resilience was spontaneously raised in all three cases studies. One interviewee noted “resilience is important in life generally, not just work” (B5) but it was identified as particularly important in systems leadership.

“You have got to keep personal resilience because we have been doing this for years now, it's not a three months push.” (A2)

The experiences shared fell into three categories: those that focus on what the individual does for themselves, those that focus on how the system works to build resilience in its leaders, and finally those that focus on how the system promotes resilience in its own structures. One interviewee described the challenges to retaining resilience and being able to judge their own needs in this area.

“I don't think I did [have enough resilience], I genuinely don't think I did. Because you are not sleeping, you're eating badly, you'd go hours without even drinking. And it is only now I am sort of coming out of it all, I realise that. You think you are fine at the time.” (N2)

Others in the Novichok system acknowledged that not enough had been done to promote a system-wide collective culture of resilience.

“One of the learning points for us out of this is that I don’t think we took that [resilience] seriously enough…. Yes, so we left it to the individuals and I think that's where we got that wrong.” (N7)

Both the ACEs and the BP case studies addressed this issue by running externally facilitated sessions to develop promoting a culture of resilience in its leaders. Building resilience at the whole system level surfaced mainly in terms of how to ensure the system could survive when key people moved on.

“People will move, and then the test is, if person leaves, is promoted, retires, what does their agency do to keep it going? Is it culturally inherent that it will continue? … Does it dissipate? Does it go away or is it embedded?” (A6)

#### People

Interviewees were asked what personal characteristics they felt were an asset for system working and which they needed to tone down. Some interviewees also talked about personal values around the work being done and the impact this has on their systems leadership approach.

##### Personal characteristics

A wide range of beneficial personal characteristics were identified. One interviewee felt this was because different parts of systems need different things.

“You could have five systems leaders in a room and they’d be different, their styles would be different but, them and their style will be fit for whatever their purpose in the system is.” (A6)

With respect to common traits, an ability to be calm under pressure, being personable, the ability to persevere, a lack of personal ego and the ability to enjoy the intellectual challenges were all raised in various ways by interviewees.

In terms of traits that interviewees felt they needed to downplay, needing to mute “the control freak” (A2) and being able to mask frustrations were two traits that surfaced in multiple interviews.

“As a [senior professional] you are constantly in charge, but for this you’ve got to try and stop yourself... from being in charge. You have got to recognise that everybody in that room is probably similar to you. You … have just got to get on with it and try and influence without being too bossy.” (B4)

Overall, being able to be flexible and manage behavioural preferences appropriately seemed more important than individual personal characteristics.

##### Values

The motivating impact of aligning personal and professional values came across as important throughout the interviews. It was striking how vocal interviewees were about their passionate belief in their work and the weight they placed on serving their population.

“You are talking from your heart. If I was trying to instil the virtues of something that I didn't believe in it wouldn't work.” (A6)

“It's written on my bones. Yes, it's what gets me up and out of bed in a morning with a fire in my belly you know.” (B7)

#### Paradoxes

Schad and Bansal [22] define a paradox in a systems context as ‘persistent contradiction between interdependent elements’, noting that such issues require an ‘an integrative (both/and) perspective’ rather than ‘a trade-off (either/or) approach’. Issues around balancing power dynamics, managing conflict and acting in an environment of uncertainty were all paradoxical issues [23] that were identified.

“It is about creating those conditions in which polarities can be managed” (E2)

##### Power

Interviewees were explicitly asked about how they felt power was distributed within the system. In all three cases studies a number of different dynamics co-existed. The polarities of command and control versus distributed leadership styles were observed along with more hybrid styles. In the Novichok scenario the command and control structures were clearly observed and legally mandated. However, other power dynamics co-existed, and leaders felt this was beneficial.

“The command and control approach to problems and the systems approach kind of clash, but they can quite happily coexist and clash, because some of this was very command and control, and had to be, and other bits were much more of a systems approach.” (N7)

Action on ACEs appeared to have the most distributed power relations, with power being shared in some cases to the community level. Interviewees from this case study commented on how rare they felt it was for power to be so widely distributed, but that it had been very beneficial.

“The good thing about it has been that we’ve resisted trying to take too much control over stuff, which, I think, is what part of the success has been due to because I think then people feel the freedom. Ultimately, people respond well when they have a sense of purpose and have autonomy to deliver that purpose.” (A4)

The necessity of being able to flex styles and benefit from using different power relations within a single system was a common theme across the case studies. It was often described in terms of balancing “autocracy and democracy” (A4). Interviewees discussed the benefits of having the versatility to move between different power relations depending on the circumstances.

“We need the balance of both… you might not want that overarching approach of command and control all the time, but actually within projects to actually get things done you do need people who are going to be like that… in the wrong setting that can be negative, but actually in the right time and place that’s job done, that’s just delivery.” (B3)

This was related to recurring calls for leaders to be flexible in their ways of working - using their power and influence in the most appropriate way to get things done. This led to some interviewees describing themselves as ‘facilitators’ rather than leaders.

“I’m not there to be the boss, I’m just there to help facilitate the collaboration.” (B10)

Interviewees noted that moving towards more distributed power relations was challenging.

“I think in statutory roles and the statutory sector we probably have too much control and we have a lot of power associated with our roles, so trying to learn not to do that was an interesting process.” (A3)

##### Conflict

It was generally accepted that tension and disagreement are inevitable in systems working. The interviewees provided several insights into how systems leaders navigate between allowing disagreements to emerge in order to provide constructive challenge, but not so far that things descended into conflict and the system was fractured.

One technique was to ensure relationships were strong enough to facilitate constructive discussion rather than direct conflict. The importance of relationships in facilitating this was described by one interviewee:

“I think it’s harder to have a big strong argument with somebody you like than it is with someone you don’t know. This is why people troll people on Twitter and stuff like that...” (B10)

A second technique identified was around framing [24]; often interviewees talked of ‘turbulence’ or ‘challenge’ as much as they did conflict. Disagreement was often framed in a positive way as ‘constructive challenge’, rather than using more negative language.

“I have to appreciate that other people have other opinions and I think as long as we can have some constructive challenge then that’s really healthy because actually it has shaped what we’re doing.” (A3)

Where there was conflict a number of guiding principles for working constructively and dynamically with it were observed. The importance of airing issues was stressed, as was a very deliberate focus on emphasising that problems were around issues and not people.

“We tried to keep the conflict professional and not personal, and really be hard on the problem and soft on the person. It's very easy to flip that the other way in times of conflict.” (N7)

However, these rules were not absolute. In one situation, a number of interviewees indicated a potential area of conflict that was deliberately not addressed because it was deemed too destabilising to work through in the time available.

“It is sometimes better just to let it go because you know that the wider group can still achieve what is needed.” (N1)

##### Uncertainty

A third leadership paradox was around balancing acting now, and risking doing the wrong thing, against waiting for better information, and doing the right thing but too late. The importance of working with uncertainty and not being overwhelmed by it was highlighted by one interviewee.

“I can sometimes be quite unsure and that can start to affect my outward appearance and how I present and how I talk. So, it's about keeping that uncertainty and fear in check as a leader and not transmitting that fear to others.” (N7)

One approach taken to living within this paradox was recognising and accepting the inherent uncertainties.

“You have to accept there's uncertainty that is the key thing… I'm relatively comfortable, in my organised way, that there will be times where it’s not black and white, it's wicked.” (N6)

A second approach was developing guiding principles for navigating the uncertainty. In the blood pressure study, they developed a system of using prototypes to test areas of uncertainty, ACEs used the principles of “Viral Change” and Novichok returned to first principles.

“We started to evolve a plan based on those basic, basic principles of health protection and public health response.” (N4)

### Indicators of success

Some indicators of when the system was working well emerged. Improved qualitative and quantitative indicators and formal plaudits were anticipated indicators of success. In contrast, the theme around the enjoyment derived from the system leadership work and the pleasure taken in the sense of camaraderie were purely inductive findings.

#### Sense of enjoyment and shared endeavour

The sense of enjoyment that interviewees derived from working in their systems was expressed very strongly both in terms of their own feelings, but also what they observed in others.

“Without a shadow of a doubt they are nicest meetings that I chair… people are not rocking up because they have to, they're rocking up because they want to.” (A7)

One interviewee discussed why enjoying the work might be both an indicator, but also a driver of success.

“At the end of the day we’re people, with emotions and all the rest of it and I think if we don’t feel good about something we’ll not necessarily gravitate towards it.” (B2)

Two factors contributing to the sense of enjoyment were identified. These were that people liked the sense of shared endeavour as well as the feeling that the work aligned with their personal and professional values.

“It's the fact that it's an important situation, that you're all working towards a common goal and that somehow mystically binds you.” (N5)

“For me the passion is personal and professional.” (A7)

#### Increased momentum with gains to system

Another indicator of success was reaching a tipping point when increased resources started to flow into the system. This tended to be after many months of work and was most comprehensively seen in the BP case study which has been in existence for longest but was also seen in the ACEs work. The additional resources most frequently identified were increased funding and increased partners and staff.

“We didn't have much for two years, and then it just eventually, you know it started to build.” (B3)

No single critical first factor was identified for attracting resource, it was felt to be cumulative.

“It's a virtuous circle isn't it, the more we do the more we get.” (B4)

In the Novichok case study the response was legally mandated and fully resourced from the outset, so this effect of increased momentum was not seen in the same way. However, the offers of mutual aid from other regions and the keenness of partners to engage were suggested as signs of a similar effect.

“People just threw themselves into it… which is a measure of something very positive.” (N1)

All three case studies reported regional, national and even international recognition of their work in terms of awards and invitations to present at conferences or feature in national best practice publications.

#### Shifts in evaluation metrics

Interviewees were asked about how they were measuring success. In Action on ACEs there was an early emphasis on collecting ‘stories’ or qualitative data to assess impact. This was done from early on. There was acknowledgment in the ACEs system that a shift in quantitative data indicators would be of increasing importance as ‘hard evidence’ of a sort understood by other organisations, was also needed. Only in the BP case study, the longest running of the three, were shifts in quantitative population indicators observed and even then it was emphasised that they could not unequivocally be attributed to the system interventions.

## Discussion

### Main findings of this study

This paper provides an in-depth thematic analysis of three UK based public health systems leadership cases studies. It identifies the systems leadership factors important in getting started with systems leadership and in maintaining system momentum. It explores the paradoxes faced by systems leaders and ways of working with them. It also touches on some potential indicators of successful systems working. Figure 1 illustrates the main findings and their relationships.

The Novichok case study had some significant differences from the other two scenarios. The incident management team is a legally mandated system and there was a clear command and control framework. At first glance this might appear in conflict with many systems leadership principles. However, the situation and actions observed were entirely compatible with the definitions of systems leadership and the concepts and theories it embodies, showing systems leadership can also be effective in situations more commonly associated with command leadership styles.

While the Novichok case study ran to a condensed timetable of a few months, the ACEs and blood pressure case studies developed over a number of years. This allowed some temporal differences in the relative importance of themes to be identified. Convening the coalition of the willing and enthusing the system with a compelling call to action appears essential in getting started, but less significant as work progresses. The ACEs and blood pressure case studies also illustrate how the significance of things like governance structures, money and qualitative and quantitative outcome indicators change with time.

### What is already known on this topic

As discussed in the introduction, there is a paucity of peer reviewed empirical research analysing *how* systems leadership is carried out in UK public health practice. There is however a range of research, policy and organisational development materials that cover more conceptual *what* and *why* of systems leadership. In a UK health and public sector context papers have come from The King’s Fund [19–21], NHS Leadership Academy [8, 25] and Virtual Staff College [9]. Internationally, Senge [26] and Seelos and Mair [27] have advanced theories in terms of practical application and the Harvard Kennedy School of Government has written on applying systems leadership principles to the sustainable development goals [4].

Often these papers are conceptual in nature, offering theoretical overviews and organising frameworks like the “CLEAR approach” [4] or suggesting ‘five important factors’ or ‘seven guiding principles’ for systems leadership. There is broad overlap in the concepts raised in these papers with relationships, engaging and energising the system in a shared sense of purpose, iterative innovation and a collective commitment to the long term being identified as important in all of them. There was good correlation between the findings from this study and the top-level themes identified above. Some concepts like The King’s Fund’s ‘coalition of the willing’ came through strongly in the interviews with the same phrasing being used by interviewees as in the original publications [20] which they may well have been familiar with.

The Virtual Staff College synthesis paper [9] offered a more elaborate and detailed model of systems leadership including the political, regulatory and organisational context, what happens at the system level and then breaking down how leaders practice systems leadership in terms of feeling, perceiving, thinking, relating, doing and being. In contrast with the approach described in the papers discussed above that focus more on what individuals do, the Ghate framework acknowledges more of the interplay between the individual, the culture and the structural context and how these factors combine to construct and sustain systems leadership. The findings from our study also position the interplay between structure, culture and people and the paradoxes that ensue as central in maintaining momentum.

There were two key concepts from the Ghate model, however, that did not emerge in this study. Firstly, the Ghate model places ‘service users’ at the heart of systems, whilst none of the interviewees in this study referred to ‘service users’, focussing instead on people and populations. Secondly, Ghate et al.’s ‘focus on product not process’ was not observed, with interviewees in this study frequently raising the importance of guiding principles around problem solving processes.

In central government literature on the UK public health system [28] systems leadership is often referred to as a key role for the local Director of Public Health specifically, rather than the wider public health profession. These cases studies illustrate that while Directors of Public Health are important, other public health professionals are also playing a key part in achieving systems leadership and creating public value [29].

### Implications for the practice of systems leadership in public health

The findings highlight a number of practical points that future systems leaders can incorporate into their practice.

Firstly, developing a compelling call to action and gathering a ‘coalition of the willing’ are both important in initiating action. These concepts translate across from the wider change management literature [30] but within a systems leadership framework they are more population and issue-focussed rather than leader-centric, and so transcend the expectation that change is initiated by a single leader.

Secondly, all three cases studies identified a need for a dedicated system coordinator role. Creating and maintain this role within a system is likely to provide a significant practical lever to ensure more effective systems leadership, a point echoed by Bolden et al. [7].

Thirdly, the findings demonstrate that relationships are essential and that while they can be built in a number of ways, consciously focussing on nurturing them is important. In terms of how to build relationships the findings show that trust, and feeling valued and respected are key. The interviewees shared their experience that this can be achieved by sharing one’s own vulnerabilities, acknowledging colleagues’ contributions and needs in a personalised way, and ensuring actions, behaviours and words reinforce a supportive and safe working environment. Routinely building such practices into their ways of working will strengthen the impact of systems leaders.

Fourth, the analysis identified that building resilience personally and structurally is essential. This means that systems leaders need to shift away from charging individuals with responsibility for their own resilience, to a more structured and deliberate approach to building system resilience. This is particularly important given how long term and demanding system working can be.

Fifth, for organisations looking to develop systems leadership it is important to recognise that ensuring diversity and inclusion is essential, as different aspects of systems working will require different forms of knowledge, expertise, experience and personal characteristics. The overall quality that was deemed most beneficial to successful systems leadership was the capacity for flexibility and adapting behaviours to fit the context.

Sixth, this study, and the wider literature [22, 31–33] emphasise that paradoxes around power, uncertainty and conflict are inherent in systems working and will be commonly faced by systems leaders. It is helpful for leaders and their organisations to understand this from the outset and accept that these challenges that are not ‘overcome’ per se, but rather that effective system leaders find ways of working constructively and dynamically with these paradoxes in order to take meaningful action and drive progress. This study suggests that recognising the issues, using guiding principles, developing confidence in operating in such environments and strong interpersonal skills can be effective approaches to working within these paradoxes. Systems leader should be supported to develop these skills and experience.

Finally, this study demonstrates the need for a wider range of indicators to be used when evaluating the effectiveness of systems leadership. A factor that was identified in all cases studies as an important indicator of success was a sense of pleasure and shared endeavour. This may be hard to measure but without it initiatives may find it hard to sustain the levels of motivation and commitment required to navigate the inevitable challenges and complexities.

### Strengths and limitations

The thematic analysis approach applied to the three cases studies allows inductive as well as deductive themes to be identified and generates accessible and practical findings. In terms of limitations, the case studies were confined to public health examples in England which may limit the generalisability of findings beyond this context. Attempts were made to mitigate this by ensuring the cases studies covered the three domains of UK public health, had a wide range of interviewees, and were examples of mainstream public health scenarios that public health professionals from many backgrounds could relate to. Bias may have been introduced due to interviewees reporting what they think they should have done rather than their actual practice. This was mitigated as far as possible by asking similar questions to all interviewees and cross-referencing responses against those of the other interviewees in the same system to build a rounded picture and identify if any positive reporting bias existed (none was found). There may also have been researcher bias that impacted on the themes identified. This was mitigated by using multiple researchers, with a range of personal and professional backgrounds, pursuing reflexivity and by consciously being open to identifying inductive as well as deductive codes and themes.

## Conclusion

This study has provided insight into the nature of public health systems leadership in a range of UK settings. It has identified empirical factors that contribute to strong public health systems leadership and has provided a diagrammatic framework to describe the elements that contribute to effective systems leadership and shown how they can be practically applied in very different public health settings. When getting started, the importance of a strong call to action and recruiting a coalition of the willing were identified. Maintaining momentum relies on the interplay of structure, culture, and people and managing paradoxes around power, uncertainty and conflict. The findings highlighted the importance interviewees placed on building resilience both personally and structurally and on having a dedicated system coordinator. The study also highlighted that interviewees in all three case studies identified an energising sense of pleasure and shared endeavour as an indicator of a system with successful leadership. Taken in combination, these findings can be used to drive improved systems leadership practice in public health.

## Supplementary information

**Additional file 1.** System Leadership Case Study Interview Schedule, Prompts used to guide interviews.

## Data Availability

The datasets generated and/or analysed during the current study are not publicly available due to the protection of individual privacy of participants. However, these and the methodological tools used may be made available from the corresponding author on reasonable request.
