# Supplementary material for: Systems leadership in practice: thematic insights from three public health case studies
Source: BMC Public Health. 2020 Nov 17;20:1735. doi: 10.1186/s12889-020-09641-1 (PMC7673088; doi:10.1186/s12889-020-09641-1)
Supplement: Supplementary file 1 — Additional file 1. System Leadership Case Study Interview Schedule, Prompts used to guide interviews. [file 12889_2020_9641_MOESM1_ESM.docx]

**
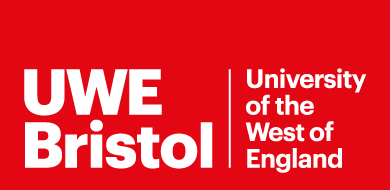
**

**DRAFT DRAFT DRAFT**

**Systems Leadership in UK Public Health Case Study Interview Schedule**

*This is a semi-structured interview schedule for the Systems Leadership in UK Public Health Case Study project.*

*Questions may need to be adapted depending on the respondent. Sub-questions can be used as prompts where appropriate and/or adapted as required. Please ensure the interviewee sees the participant information sheet and completes the consent form before the interview.*

*If interviewees raise any queries/concerns about the study please refer them to Professor David Evans or Dr Charlotte Bigland.*

**BACKGROUND**

1. **Interviewees background and current role?**
   1. *Role/purpose of organisation*
   2. *Experience in public health*
   3. *Relationship to the wider system of UK public health*
2. **Understanding/experience of systems leadership?**
   1. *What do you understand by it?*
   2. *Does it matter?*
3. **Overview of the project that will form the focus of this case study?**
   1. *Who was involved?*
   2. *Key events/actions*

**AIM**

1. **“Higher purpose”**
   1. Goal project is trying to achieve
   2. Professional and/or personal feelings about this

**PROCESS**

1. **Getting started - Approach to getting strategic buy-in**
   1. Variation in approach?
2. **Relationships**
   1. How formed, with whom?
   2. Different to other professional relationships? Value?
3. **Power Dynamics**
   1. Where was power held?
   2. Shift with time?
   3. Control vs influence?
   4. Distributed power?
   5. Formal TOR?
4. **Risks**
   1. Organisational risks taken?
   2. Personal risks taken?
5. **Innovation/experimentation /disrupting system**
   1. What different to normal
6. **Conflict**
   1. Overt or hidden?
   2. How addressed

**CONTEXT**

1. **Political, regulatory, financial, local**
   1. Decision making power – red tape?
   2. Sufficient operating resources – people/finances
   3. Public value recognised widely
   4. Political support
   5. What were the enablers, what the barriers?

**PERSONAL ATTRIBUTES/STYLE**

1. **Describe your personal approach or mindset?**
2. **What personal qualities and behaviours did you amplify, which did you mute?**

**CLOSING**

1. **Do you have any final thoughts/comments on system(s) leadership in public health you would like to share before we finish the interview?**
